# The last one heard: the importance of an early-stage participatory evaluation for programme implementation

**DOI:** 10.1186/s13012-014-0137-5

**Published:** 2014-09-26

**Authors:** Brynne Gilmore, Frédérique Vallières, Eilish McAuliffe, Nazarius Mbona Tumwesigye, Gilbert Muyambi

**Affiliations:** Centre for Global Health, Trinity College Dublin, 7-9 Leinster Street South, Dublin 2, Ireland; School of Public Health, College of Health Science, Makerere University, P.O. Box 7062, Kampala, Uganda; World Vision Uganda, Kisozi Complex, P.O. Box 8759, Kampala, Uganda

**Keywords:** Maternal and child health, Community health workers, Participation, Evaluation, Programme implementation, Project cycle

## Abstract

**Background:**

The systematic involvement of project beneficiaries in community maternal and child health programmes remains low and limited, especially during the formative stages of the project cycle. Understanding how positive and negative feedbacks obtained from communities can subsequently be used to inform and iterate existing programmes is an important step towards ensuring the success of community health workers for maternal and child health programming and, ultimately, for improving health outcomes.

**Methods:**

The study took place over a period of 4 weeks in North Rukiga, Kabale District of southwestern Uganda. Using a cross-sectional qualitative study that employed an epistemological approach of phenomenology, nine focus group discussions and eight in-depth interviews were conducted with a total of 76 female participants across six different sites. Women were identified as either users or non-users of the maternal and child health programme. Purposeful sampling was employed to recruit women from six different locations within the programme catchment area. Translated and transcribed transcripts were subjected to a bottom-up thematic analysis using NVivo 10 Software, whereby themes were arrived at inductively.

**Results:**

Predominant themes emerging from the focus groups and key informant interviews identified early trends in programme strengths. Beneficiaries reported confidence in both the programme and the relationships they had forged with community health workers, exhibited pride in the knowledge they had received, and described improved spousal involvement. Beneficiaries also identified a number of programme challenges including barriers to adopting the behaviours promoted by the programme, and highlighted issues with programme dependency and perceived ownership. It also emerged that community health workers were not reaching the entire population of intended programme beneficiaries.

**Conclusions:**

This research provides support for the importance of an early-stage participatory evaluation of beneficiaries’ perceptions of newly initiated health programmes. Our results support how evaluations conducted in the early phases of programme implementation can provide valuable, timely feedback as well as yield recommendations for programme adjustment or re-alignment, and in turn, better meet end-user expectations. Potential reasons for the observed lack of community participation in early stages of programme implementation are considered.

## Background

A growing body of literature advocates for the important contribution of formative evaluations in the implementation of health programmes [1,2]. These early-stage evaluations provide in-depth, contextually relevant, and timely information on factors that can potentially enhance or impede implementation success. However, formative evaluations are rarely utilised, or if they are, seldom are they documented for peer review. What emerges is an increasing need to report methodologies for conducting these evaluations to ensure their findings are applied to practice [1,2]. In addition, and despite the Paris Declaration on Aid Effectiveness’ recommendation to increase the use of local systems for programme design, implementation, and monitoring and evaluation, local involvement in health programmes remains low and limited during the formative stages of a project cycle [3,4]. An overemphasis on measuring clinical outcomes, most of which are aligned to the indicators outlined in the Millennium Development Goals (MDGs), has led to the neglect of other important factors. Namely, the key characteristics and processes through which effective outcomes are achieved and the opinions of the end-users with regard to how effective they perceive the intervention to be [5].

Progress towards meeting the MDGs 4 and 5 remains particularly slow in under-resourced areas of low- and middle-income countries (LMICs), where 99% of maternal and child deaths occur [6]. Each year, a quarter of a million women die from complications related to pregnancy and childbirth and 7.2 million children die before they reach their fifth birthday [7,8]. Faced with these stark figures, most countries are not expected to meet MDGs 4 and 5 by 2015 [7,8]. Uganda is one such country [9,10]. The most recent Ugandan Demographic Health Survey reported a maternal mortality ratio (MMR) of 438 per 100,000 live births and an under-5 mortality of 90 per 1,000 live births [11]. The primary causes of child mortality in Uganda remain malaria, neonatal diseases, pneumonia, and diarrhoea, comprising 25%, 23%, 19%, and 17% of child deaths, respectively [12]. Apart from the high under-5 mortality rates, there are many other areas of concern for child health in Uganda, with 32% of children under-5 being stunted [13]. Seventy-three percent and 20% of children under-5 in Uganda are iron and vitamin A deficient, respectively [12]. Only 52% of children between 12 and 23 months are fully immunised, falling short of the 80% target [11]. Despite neonatal deaths accounting for 39% of all infant deaths [14], 86% do not receive any postnatal visit, with only 2% of newborns receiving care within the recommended first hour after birth and only 9% receiving care within the first 24 h [11]. The 2011 Demographic Health Survey also identified appropriate breastfeeding practices for children 0–24 months as problematic, as only 63% of children under 6 months are exclusively breastfeed, and only 6% of children 6–23 months are fed according to Infant and Young Child Feeding (IYCF) recommendations [11]. As a result, Uganda continues to rank among the world’s countries with the poorest maternal and child health indicators.

Evidence-based, cost-effective interventions for reducing maternal and child morbidity and mortality are well known throughout the literature [15,16]. When implemented at the community level, these simple interventions are estimated to prevent between 40% and 72% of under-5 deaths [17,18]. Most of these interventions hinge on specific changes in health behaviour and include practices such as accessing health care and vaccinations at appropriate times, exclusive and appropriate breastfeeding, sleeping under an insecticide treated net, and improved hand-washing and water storage behaviours [19]. Unfortunately, the low adoption of these practices among both women and children has significantly hampered their success in improving maternal and child health outcomes [20]. The uptake of these evidence-based interventions is often hindered by a lack of early-stage engagement of the very communities programmes are meant to serve [21]. What emerges too often is a system where programmes are dictated by top-down processes, which, some argue, shifts the ownership and accountability away from communities and beneficiaries [22]. Results from previous RCTs in Uganda, for example, demonstrate that strengthening community monitoring and accountability mechanisms can significantly increase both health outcomes and service utilisation [23]. Another second factor contributing to the low coverage of effective community-level interventions is a global shortfall of approximately 4.3 million health workers [24,25]. Community health workers (CHWs) [26] have the potential to act as an important bridge between communities and formal health systems [27] and are effective in providing essential MCH services during the critical days surrounding childbirth for both mother and baby in resource poor settings [28-33].

Understanding how positive and negative feedback can be obtained from communities and how such feedback can subsequently be used to inform and iterate existing programmes is a crucial step towards ensuring the successful adoption of MCH programming. Changes in health behaviours should be achieved through incremental improvement and the incorporation of learning through community participation [21]. Community participation, interchangeably used here with engagement, is considered as the process through which community members influence CHW for MCH programmes, with the goal of enhancing community well-being [34]. Though several challenges for its practice exist [35], community participation is recognised within Alma-Ata as best practice in health programmes and has been linked to increasing ownership and quality of services, which can subsequently impact on the success of activities [36-38].

An effective method of promoting community participation is through the involvement of programme beneficiaries throughout the monitoring and evaluation (M&E) process. Exploring the factors that contribute to the success and/or failure of community members, such as CHWs, engaging in MCH programmes provides an important opportunity for shared learning on what policies and implementation strategies are most effective in the improvement of MCH outcomes at community level. By studying users’ views of a health service, it becomes possible to identify areas for improvement and strengthening as well as acceptance of the initiative. Involving project beneficiaries in the evaluation of programmes can enhance individual self-determination [39], empower individuals [40,41], and assist in addressing inherent power imbalances between project beneficiaries and staff [42]. Atkinson and Haran [43] state that assessing user satisfaction can increase compliance, explore knowledge transfer and patient involvement, inform policy design, and compare alternatives. Furthermore, user satisfaction can predict acceptance of a proposed strategy, which often influences future utilisation, and compliance of care [44,45].

Understanding a health service from the user’s point of view has important and widely applicable implications for the future implementation of CHW for MCH programmes in Uganda and for the broader public health policy arena. The purpose of our research is to better understand mothers’ perceptions and acceptability to a newly initiated CHW for MCH strategy at the initial stages of programme implementation through a participatory formative evaluation. By conducting this type of investigation, it is anticipated that the beneficiaries become an active part of the monitoring and evaluation process. Moreover, the implication is that findings can potentially contribute to inform programme iteration for health-care policy and decision makers from an early stage so that appropriate, timely changes can be made. Finally, a greater understanding of the perceptions of target beneficiaries may lead to specific changes that make the programme more acceptable, therefore increasing utilisation and ultimately improving MCH outcomes. This research is timely given an increasing demand for a shift in methods and standards of evaluation. Programmes are increasingly moving from output evaluations to the evaluation of results and impact, while developing more collaborative approaches to programme evaluations [3,40].

### 7–11 timed and targeted counselling (ttC)

The research described focuses around a newly initiated maternal and child health programme, Access to Infant and Maternal Health (AIM-Health). The AIM-Health employs a behaviour change communication (BCC) approach to improve health and nutrition outcomes for pregnant women and children under-2 through the use of timely and appropriately targeted messages. These messages originate from evidence-based, cost-effective practices [15,16], and are highlighted in both WHO and UNICEF guidelines as best practice to target the primary causes of maternal and child morbidity and mortality in LMICs [46]. The messages focus on *7* key interventions for pregnant and lactating mothers and *11* key interventions for children under-2, which is known as “7–11” within World Vision’s programmatic approach. The 7–11 interventions are subsequently delivered through a minimum of ten household counselling visits made by a CHW at specific times. The counselling approach is based on the American College of Nurse-Midwives home-based life saving skills (HBLSS) technique [19].

## Methods

### Study setting

This study took place over a period of 4 weeks in North Rukiga, Kabale District of southwest Uganda. This area was chosen since it was approximately 2 months into a newly initiated 5-year CHW programme led by World Vision Ireland. The AIM-Health programme is currently being implemented across ten settings in five different sub-Saharan African contexts. In Uganda, the programme is run in collaboration with World Vision Uganda and the Ugandan Ministry of Health (UMOH). All research occurred in the North Rukiga county of Kabale District in southwest Uganda, which consists of primarily of subsistence farmers and comprises two sub-counties, Kashambya and Rwamucucu, totaling 13 parishes. The southwest region of Uganda has some of the worst MCH indicators for the country, with only 25% of deliveries occurring in a health facility, and 19% of women giving birth with no assistance whatsoever [11]. This region also has the lowest percentage of postnatal care (PNC) with only 18% attendance [11].

### Study design

We conducted a cross-sectional qualitative study that used the epistemological approach of phenomenology through in-depth interviews (IDIs) and focus group discussions (FGDs) to understand a woman’s lived experiences with the CHW programme to date. Village chairpersons acted as mobilisers using purposive sampling to gain access and disseminate research materials to potential participants.

### Data collection

A semi-structured interview guide was developed for both FGDs and IDIs to assist in eliciting individual responses; however, it was not followed rigidly as phenomenological research is concerned with experiences and issues of importance to the individuals and is based on personal knowledge and subjectivity [47]. Women were given a preference of attending an FGD or an individual interview. FGDs are recognised as a strong methodology when discussing potentially sensitive topics [48], and the researchers felt this method may help women be more forthcoming about both their pregnancy experiences, as well as any problems they were facing with the MCH programme. Individual key-informant interviews were also offered as a methodology since using both FGD and in-depth interviews increases comprehensiveness in collection and allows for a more reflexive analysis [49].

Participants were first asked a question not specific to the programme (i.e. “What are some of the challenges that pregnant and nursing women face in your community?”). During the interview, the researcher encouraged the participants to control the flow of the conversation. Though attempts were made to avoid direct questions, the researcher was required to ask additional probing questions when conversations stalled. Interviews were conducted in the local language (Rukiga) by a female health professional, trained in interview techniques, and who was familiar with the AIM-Health programme. A member of the research team was also present to capture the tone, mannerisms, and note the body language of the participants. Recorded discussions were subsequently transcribed, translated, and the content verified by a bilingual Rukiga and English speaker. In total, nine FGDs and eight in-depth interviews were conducted with a total of 76 participants across six different sites, three from each of the sub-counties (Table 1).

**Table 1:** **Data collection location and response**

**Site number**	**FGD no. of participants**	**IDI no. of participants**	**Users**	**Non-users**
1	FGD1 = 6	N/A	14	0
	FGD2 = 8			
2	FGD3 = 7	IDI = 3	10	0
3	FGD4 = 7	N/A	7	0
4	FGD5 = 7	N/A	21	0
	FGD6 = 5			
	FGD7 = 9			
5	N/A	IDI = 5	5	0
6	FGD8 = 9	0	2	17
	FGD9 = 10			
Total	*n* = 68	*n* = 8	*n* = 59	*n* = 17

### Study participants

Participants were pregnant and/or breastfeeding women residing in World Vision’s programme area of North Rukiga and therefore potential beneficiaries of the AIM-Health project. Women were self-identified as either users or non-users of the service provided by CHWs as part of AIM-Health, had to be at least 18 years of age, and had to have completed the informed consent process. Women were considered “users” of 7–11 if they had been visited at least once by a trained CHW. Contrastingly, a “non-user” was defined as a woman who had not been visited by a CHW trained as part of the 7–11 strategy despite living in the programme target areas. Purposeful sampling using community “mobilisers” was employed to recruit only AIM-Health potential beneficiaries from six different locations within the programme catchment area. Community mobilisers included either pregnant women or community nurse/midwives working out of a local health facility. Mobilisers were given participant information leaflets to either distribute or read to potential participants in their communities 7 days prior to data collection, which included information on participant requirements, methodology, and purpose of study. Interested participants were asked to reconvene at a particular date and time if they wanted to participate in the study. Specific times were allocated for both the FGDs and IDIs, with participants choosing which time and therefore which interview method they wanted to participate in. Upon arrival at the study site, the self-identified as either pregnant and/or breastfeeding women were asked their age and were asked for their preference of type of interview type. The six different locations were identified in consultation with UMOH officials, who chose them as they felt these locations best represented the population of North Rukiga.

### Data analysis

Translated and transcribed data were subjected to a bottom-up thematic analysis using NVivo 10 Software. No thematic groups were determined prior to data collection and analysis. A “theme” was therefore characterised as a recurrent underlying concept that offers a general insight from the entire data range [50]. Themes were arrived at inductively, as no hypothesis or thematic groups were present prior to analysis [47]. The analysis process was continuous and relied on the researcher’s growing familiarity with the data [51]. The analytical approach used was constructivism whereby the researcher employs a transactional and subjective approach and uses the interaction between participant and herself as well as her own personal knowledge and experience to analyse the data [51]. All transcripts were read thoroughly, and two main thematic groups (positive experiences and negative experiences) were included in the first level of coding. These groups were subsequently coded further to form sub-groups, with a third level of analysis occurring in specific themes that were rich in data. Though the research team attempted to reduce preconceptions and individual convictions during the collection and analysis phase, phenomenology and constructivism both recognise that analysis is a cognitive process and as such, it is subject to the researcher’s own view, understanding, conceptual orientations and experiences of the world, and is therefore undoubtedly affected by what they witness [51,52].

### Ethics

Ethical approval for this research was obtained from the Health Policy and Management/Centre for Global Health Research Ethics Committee, Trinity College Dublin and the Higher Degrees Research and Ethics Committee, Makerere University School of Public Health, Uganda. Aligned with best practice for illiterate participants, both written and verbal informed consent was obtained from participants.

## Findings

### Participant characteristics

A total of 76 pregnant or breastfeeding women participated in this study, of which 17 had not been visited by a CHW despite living within the programme catchment area despite having previously expressed a desire to be enrolled (Table 1). The majority (*n* = 70) was unemployed and only 23% had at least some secondary education. Seventy-two participants reported attending at least one ANC visit, with 2 non-users and 2 users having never sought ANC services. The mean reported walking time to a health facility of was 75 min (*s* = 76.6). Twenty-five participants reported living over 1.5 h walking distance from the health facility, despite the acceptable distance of 5 km (assumed to take approximately 1-h walking), as defined by the Government of Uganda [13]. For 70 (92.1%) of respondents, the nearest health centre was a Health Facility II, which is not equipped with ANC or delivery services. Table 2 includes demographic information for all study participants. The perceptions of both users and non-users towards the MCH programme highlighted areas of encouragement regarding the programme, some unexpected initial trends, as well as areas of concern that if not addressed, may hamper the programme’s success.

**Table 2:** **Background characteristics of participants (** ***n*** **= 76)**

**Characteristics**	**No. of respondents**
Age range (years)	18–43
Mean age (years)	25.5
Number of pregnancies	
1	11
2	17
3	20
4	6
5+	22
Education	
None	4
Some primary	23
Complete primary	31
Some secondary	9
Complete secondary (S4)	5
Trade school	1
Other	3
Employment^a^	
None	70
Trade/shop work	2
Wage employed	4
Distance to health facility (min)^b^	
0–30	29
31–60	21
61–90	1
91–120	16
121–150	1
150+	8
Average distance to health facility (min)	72

^a^None was participant identified, though most women in the area participate in daily agricultural activities for household consumption and/or to sell.

^b^70/76 women identified a Health Facility II as their closest facility; however, no ANC or delivery services are available at these centres.

### Programme strengths

Predominant themes emerging from the focus groups and interviews that highlighted early trends in programme strengths were as follows:

#### Confidence in and importance of CHWs and 7–11

The value ascribed to both the work of the CHWs and the MCH programme initiatives was high for all (users and non-users), as they identified the potential benefits to both their own and their families’ health. When asked what changes were noticed since the initiation of the programme, user participants identified a number of positive changes ranging from improvements in malnutrition to household water and sanitation. As one respondent states:

“*We didn’t have a toilet, I see my man is trying to construct one. We didn’t have a bathroom, I see the man is trying to build one. We didn’t even have time to sweep. I see I’m now trying to sweep.*” (FGD2, 37, user)

In addition, women viewed the CHWs as agents of change that are able to impart knowledge that communities would absorb and explained that these would then have positive health impacts. As one non-user described:

“*The change which would come, like those women who deliver from home, like when they [the CHW] tell them that they have to go to the hospital people would deliver from the hospital, but because some of them have not been told, they might get problems which they would not have got.*” (FGD9, 27, non-user)

#### Pride in health knowledge

Throughout the interviews, feelings of pride and empowerment in one’s health knowledge and ability to carry out the AIM-Health programme were evident. Observation methods used during the focus groups noted that women began to act as teachers to other participants, passing on recently learned health messages with confidence and enthusiasm. When discussing the MCH messages delivered by CHWs, women who were enrolled in the programme often went into great detail about maternal and child health care practices. On more than one occasion, when the women were asked if there was anything they wanted to add, they spontaneously took turns displaying their knowledge of MCH, in an obvious attempt to display what they had learned. In contrast, women who had not been visited by the CHWs did not exude the same level of confidence around pregnancy-related issues and often doubted their abilities, indicating that they needed assistance and requesting future visits from CHWs.

#### Relationship with CHW

In general, the CHWs that visited women in their homes were highly regarded due to their knowledge, commitment to the job, and their caring nature. The consistency of care offered by the CHW was made apparent when one participant stated:

“*These doctors in our village, I see we have a very good relationship with them, a very good relationship with them, because they keep on coming to see how our health is…*” (FGD2, 30, user)

Several enrolled participants also identified and recognised situations where CHWs undertook activities that went beyond their job description, for instance supplying food or paying for transport to the hospital, which appeared to further strengthen the bond between the CHW and the mother. One woman exemplifies this when she explains how she sequestered CHWs when dealing with marital disputes, stating:

“*I tell them [CHWs] that ‘for sure, my man is beating me seriously’ , or sometimes I tell them he wants to beat me up. They came and cool him down.*” (IDI, 22, user)

#### Spousal support

Increased male involvement, as previously seen in the case of increased participation in the construction of latrines, was a reoccurring theme throughout the discussions. Increased spousal support emerged specifically from women who received home visits by CHWs, as they commonly expressed changes they had noticed in their husband’s behaviours towards their pregnancy and childcare:

“*Since this programme came a man would just impregnate you and go but now when you know that you are pregnant, even if it is like one month, he plans to go with you to the hospital and you continue to go for antenatal care with him.*” (FGD4, 22, user)

Participants expressed satisfaction with these changes and attributed increased spousal involvement and knowledge to the CHWs involving both them and their husbands in the counselling sessions. Statements like the ones below were common and present in all focus groups and interviews with women who were enrolled in the programme:

“*But when they [the CHWs] went for training, when they [CHWs] find you at home with your man, they teach you and you see the man listening to everything and making sure he does it.*” (FGD7, 24, user)

Interestingly, women who were not enrolled in the programme commonly brought up issues around their partners’ lack of involvement in or understanding of her pregnancy. Women often acknowledged that they believed being visited would change their husbands’ attitudes towards their pregnancy, as one woman stated:

“*…Because they [the CHWs] didn’t come to advise us, when they [husbands] are in our homes with us, it brings a problem very much because when you tell him [the husband] that, ‘this and this is needed’ , like, if a woman is pregnant, she doesn’t need to carry heavy things, he will just look at you, as if he has not heard anything.*” (FGD8, 20, non-user)

### Programme challenges

Disconcerting themes identified through the focus groups and interviews with both enrolled and non-enrolled women are as follows.

#### Barriers to programme adherence—insufficient resources and hospital level

The most predominate theme in this category is that the project did little to reduce the barriers that impede women from engaging in appropriate and programme-encouraged health-seeking behaviours. Women questioned to what degree knowledge alone could contribute towards the improvement of health outcomes for their families. This was a common theme, which featured strongly in every focus group and all but one in-depth interview. Two main barriers, a lack of financial means and resources and barriers at the point of the health centre, were cited as contributing to the inability of women to engage in the healthy behaviours promoted by the CHW. Securing resources to procure materials and food proved very difficult for many women. As two women stated:

“*I saw them coming to advise us when we have given birth, that you should eat well, like meat, yet you don’t have money… We don’t have a way of getting the things they are telling us.*” (FGD4, 32, user)

“*If they advise me on something which I can manage to do, I’m O.K. But if they are advising me on something which I can’t do, I feel bad.*” (FGD7, 20, user)

Women also met barriers to a healthy delivery at the health centre level. This appeared to be especially frustrating for mothers. Issues such as not having their own delivery kits, having no money for transport in the case of a referral, and either the lack of health staff or quality of care received were all identified as barriers encountered at the health centre. Many of these barriers were mentioned by both users and non-users of the programme, with non-users citing husbands as an additional barrier.

#### Ownership

Though the AIM-Health programme intends to be a community-based and community-led initiative, overseen by the Ministry of Health and only supported by World Vision Uganda, women almost exclusively referred to it as a World Vision programme. Commonly cited notions, such as “*This programme of World Vision…*” (FGD6, 32, user) or “*These doctors [the CHWs] in the village you have given us…*” (FGD2, 37, user), highlight the fact that despite best intentions, community members do not regard the programme as the community-led initiative. A potential repercussion of this lack of ownership is demonstrated when a non-user expressed that though there was a problem with the CHWs not visiting homes, she was unable to impart change, stating, “*It makes us feel bad, but we have nothing to do about it.*” (FGD8, un, non-user).

When the researcher specifically asked several non-users what they recommend as a possible solution to this situation, many stated that it was World Vision’s responsibility to remove the inactive CHWs and to select and train new CHWs to fill their role.

#### Dependency

AIM-Health intends to improve MCH outcomes though capacity building and empowerment, and refrains from providing service delivery; however, another emerging theme was the reliance on the programme to provide essential tangible resources such as Mama Kits (local delivery kits which include soap, razors, gloves, plastic sheeting, etc.), medicine, and bed nets. Though women did value the knowledge imparted to them by the programme, it was often undermined by the lack of resources they had available, which one participant stated, should be supplied to them:

“*For sure, if they only give us advise, when the time reaches you don’t have anything in your hands.*” (FGD7, 41, user)

#### Lack of understanding of the roles and responsibilities of the CHWs among participants

Both users and non-users were familiar with the AIM-Health programme active in their communities and due to the high visibility of this programme, came to expect that CHWs would visit the households of pregnant women. However, participants frequently had misinformation, or a lack of information, on the roles and responsibilities of the CHWs. This included misunderstanding that the CHWs act as volunteers and that they receive no financial compensation for their work, the expectation that CHWs could dispense medication as part of their remit, and for non-users, where to report a non-active CHW in their community. The research team was frequently asked to explain the role of CHWs as well as the programme itself.

## Discussion

The popular view that increased user satisfaction can contribute to health service effectiveness should provide sufficient reasoning for both its investigation and its function as a method of early programme evaluation [43-45]. Despite this however, user perceptions of health services in LMICs are rarely addressed and even more rarely incorporated into programme iteration procedures [53]. Our results provide additional support for the importance of involving end-users and project beneficiaries from the very early stages of project implementation. The feedback obtained from such an evaluation not only serves to highlight a programme’s strengths and weaknesses at what, in theory, is a more flexible point in the project but can also identify unexpected consequences, which can be further monitored throughout the project cycle.

Highlighted in Alma-Ata as a key principle of Primary Health Care for All, community participation must be continuously promoted to encourage active and reflective participation throughout the project cycle, especially in the early stages of project implementation. It is our view that community-based approaches for MCH require participant involvement and feedback from the early stages of programme implementation and that user perspectives of implementation should be a standard M&E reporting indicators. Making such evaluations standard allows for a more timely reflection on user views of service during the implementation phase [54] while promoting the involvement of the community throughout the project cycle. Though the appropriate timing of such evaluations is debated within the literature [55], this paper argues that when attempting to understand users’ perspectives of programmes, evaluations can and should be conducted soon after programme deployment. Though similar evaluations will differ depending on their appropriateness to context, they require flexibility to capture the complex interactions that occur within health promotion programmes [56]. Our research demonstrates that such evaluations should occur early after the initialisation of programmes, as potential users often require little exposure before forming an opinion that can have lasting effects on long-term use.

### Participant characteristics

In Uganda, as in many other LMICs, living within lower wealth quintiles and not having attended any secondary school makes one more vulnerable to poor maternal health care practices [11,57]. The reported ever use of ANC services in the study population is comparable to that of the Ministry of Health 2010 statistics of 95% [11]. A more concerning demographic is the reported travel time to a health facility, an average of 75 min. The fact that the majority of nearby health centres do not provide ANC services indicates that families must travel even farther for MCH care and highlights the importance of CHWs or other outreach services in the area.

This early-stage evaluation also revealed that not all intended programme beneficiaries were being reached by CHWs. Potential participants were required to be residing within the programme area and to be pregnant and/or breastfeeding; they were not required to be programme recipients. The possible situation of non-active CHWs in some programme areas is concerning and may indicate a potentially larger issue with the overall programme structure. In this study, all non-users were located within the same sub-district, and these results were fed back to World Vision promptly as to address this service gap and provide care for all women residing in the area. Though CHW programmes have been identified as a means to increase health coverage and have been shown to be effective in MCH programmes [29-32,58], prevalent issues common to many CHW programmes that can impact their success and sustainability are well documented and should be considered during initiative development [59-62].

### Strengths of the programme

The CHWs as part of the AIM-Health programme were trusted in North Rukiga, with women expressing confidence in the knowledge and messages relayed to them. Users and non-users indicated that community health workers could bring positive changes to the health behaviours of both women and men. Future formative evaluations of similar programmes may benefit from conducting additional FGDs with male, or “husband”, participants. Recognising the importance of a CHW’s work, as well as the perception that without the CHWs individuals may have more negative health behaviours, highlights the value placed on such work. Viewing your health worker as knowledgeable can increase one’s satisfaction and utilisation of a service [63]. Specifically in Uganda, a user’s perception of their health worker and their training has been shown to increase acceptance and use of service [64-66]. The positive attributes ascribed to the health workers and the programme’s initiatives as well as their overall acceptability should assist in keeping women enrolled in the AIM-Health programme, and following the teachings of CHWs during the recommended timeframe, which it can be inferred, will subsequently increase health outcomes for both mother and child.

The pride the women took in their health knowledge may aid in empowering women, making them more confident in their ability to practice health behaviours and hence more willing to adhere to the programme messages and more likely to encourage other women to participate. Understanding how one’s pride in their own abilities, perhaps as a form of increased self-efficacy, may impact health-seeking behaviours or even knowledge dissemination, and what effects this may have on MCH outcomes should be further explored. Though it is unclear if the levels of knowledge on MCH practices differ between users and non-users, there was a clear difference in the level of confidence exuded by the two different groups when it came to exhibiting their health knowledge. Similarly, differences were observed in the women’s willingness to express and share the knowledge they had acquired thus far as part of the programme. The relationship between the CHWs and the women may change the dynamics of the CHW’s perceived responsibilities, as some participants appeared to ask CHWs to weigh in on issues beyond the scope of their training and task description.

The strong relationship with the CHWs as identified by enrolled women transcends the typical patient-provider role and can serve to impact satisfaction of service and subsequent utilisation of services [63]. These strong relationships may be attributed to a positive perception of the CHWs due to their perceived knowledge, their ability to relate to CHWs, and the frequency of visits (minimum ten) that encourages the development of a relationship. On the positive side, this may increase job satisfaction and retention for CHWs, whereby feeling appreciated and having respect and commendation from community members are important motivators for CHWs [61]. On the other hand, CHWs may sometimes feel obliged to provide additional resources, such as food or transport costs, to households out of their own pocket. Not only can they often not afford these resources themselves, but the expectation that they would provide for these extends far beyond their remit. Lastly, there is a chance that CHWs may be asked to become involved in a situation they are ill prepared to handle, such as the case of domestic abuse (something that some of the women indicated they had discussed with the CHW).

The trend of women ascribing increased partner involvement in MCH to the CHWs was an unanticipated outcome of the health programme and further highlights the importance of conducting an early-stage evaluation assessment. Such issues can now be followed up, researched or strengthened through changing programme activities that will more actively and consistently support this important aspect of delivering MCH services. Male involvement in MCH is recognised as beneficial to promoting positive health behaviours and outcomes [67,68]. This is consistent with past research in Uganda, which found that males who were knowledgeable on ANC, and who had received information from health workers, were more likely to accompany their partner to ANC visits [69]. Though recognised as an area with a dearth of research [70,71], other studies conducted in Uganda have called for more attention to be paid to the role of male head of households (as primary decision maker for the family) and how they may mediate access to MCH services [72]. Taken together, male involvement in MCH services in Uganda remains an important factor to ensure the successful implementation of MCH interventions.

### Programme challenges

Community ownership of CHW programmes is widely recognised as an indispensable element to success and sustainability [28] and also has implications for CHW attrition and motivation by increasing support by and accountability to the community [61]. Both users and non-users of the MCH programme seemed unaware that the community health workers are accountable to the communities in which they serve, who are in turn, are ultimately responsible for their selection or exclusion. A lack of ownership is not only in opposition to the fundamentals of community-based health programmes but it also threatens its impacts and future directions and the ability to deliver quality community-level care at scale. The choice of words that many women used to describe the CHWs such as “doctors” may signify several things, including the trust in the CHWs’ abilities and also, more worryingly, a level of power or authority. Further investigation into whether this term was intended to ascribe faith in CHWs, or was developed out of systems of hierarchy and power, and how this may impact relationships could further complement this work. Dependency on the programme to provide tangible products, for example, food, clothes and birthing kits to assist in facilitating a healthy pregnancy, childbirth and early child development years, is concerning as it can impact the sustainability of the programme and is not within the programme’s current mandate. This level of dependency and the expectation of product deliverables also highlight a lack of programme awareness and allude to barriers women face in fulfilling the recommendations by the CHW.

Even when women recognised the importance and expressed a desire to adhere to the programme’s recommendations, they are often unable to fulfil them. Women frequently cited barriers they faced in achieving healthy MCH practices, which are consistent with common barriers sited throughout the literature [73-75]. These barriers, at the clinical, outreach, and community levels, have been shown to reduce MCH care uptake throughout the continuum of care for mother and child. More extensive reviews of such barriers are covered elsewhere in the literature [73,74]. Though many of these barriers could be addressed through supply-side instruments, as this programme is not meant to be a service delivery mechanism, other strategies need to be developed to reduce the influence these have on women and children’s capabilities to access care. Inadequate programme understanding or information contributes to a lack of ownership and dependency on the programme. In addition, other areas of concern might be reduced if community members’ expectations were modified through a better understanding of the programme structure, intentions and activities.

### Implementation implications

The findings from this research and their timely dissemination to both the Ministry of Health and World Vision allowed for changes in implementation to reflect the views of programme users. A policy report resulting from this research was distributed to nine other AIM-Health Programmes running in five sub-Saharan African countries, so project implementers were aware of the challenges for this particular context. As previously stated, an area identified with non-active CHWs was targeted for further training or selection of new CHWs and the importance of spousal involvement in the counselling sessions was emphasised throughout refresher training for CHWs and in local health centres in North Rukiga. Follow-up with World Vision staff has noted that spousal involvement in ANC and PNC continues to increase in the region. Several of the main barriers that women identified throughout the research were also considered for implementation. This includes the plan for motorcycle ambulances for pregnant woman and children, which has been initiated to assist in reducing barriers of transportation in emergency situations, as well as increasing essential delivery materials in health centres. The programme has also scaled up their nutrition interventions, recognising under-nutrition as a major contributor to morbidity and mortality in the area, as well as the multiple barriers families face in providing adequate nutrition for children under-2.

### Limitations

The authors acknowledge that this study is not without limitations. As with most interview methods, there is a possibility of social desirability and respondent bias. Though appropriate measures were taken to ensure the authenticity of the data, some may have been lost in translation as IDIs and FGD were not conducted in the primary researcher’s native language and required translation. Characteristics of both the lead researcher and translator may be considered a limitation, as the researcher was not from the area and participants may have interpreted both as coming from positions of power.

## Conclusions

This article reports on the investigation of user perceptions of a newly initiated MCH programme in southwest Uganda that delivers evidence-based interventions through community health workers. Our results exemplify how early-phase evaluations can serve to highlight programme strengths and expectations and also unveil previously unanticipated consequences of programme implementation that merit further investigation, follow-up, and programmatic support. This research contributes to the existing literature on formative evaluations and the need to further develop these methodologies for conducting such evaluations to ensure their findings are applied towards the iteration of existing health programmes and policy. Furthermore, this study supports the need for early-stage formative evaluations of beneficiaries’ perceptions towards health programmes as an appropriate community participatory and evaluation method that should be integrated into standard M&E reporting processes, an approach that is too frequently omitted in development projects. The reasons for the observed lack of community participation might lie, in part, in the increasing pressure on NGOs and their implementing partners to prove their value-add, resulting in their accountability focus being more towards donors rather than beneficiaries, and from a concern that funding implementation research may divert funding from NGO core programming activities. Amidst the static of rigid programming, complicated reporting structures, imposed donor agency ideologies, and frequent top-down approaches to “development”, the community’s voice is too often the last one heard.

## Abbreviations

AIM-Health: Access to Infant and Maternal Health
ANC: antenatal care
BCC: behavioural change communication
CHW: community health worker
LMIC: low- and middle-income country
MCH: maternal and child health
MDG: Millennium Development Goal
M&E: monitoring and evaluation
MMR: maternal mortality ratio
MOH: Ministry of Health
PNC: postnatal care
